# Vascular Endothelial Regulation of Obesity-Associated Insulin Resistance

**DOI:** 10.3389/fcvm.2017.00051

**Published:** 2017-08-09

**Authors:** Manna Li, Ming Qian, Jian Xu

**Affiliations:** ^1^Department of Medicine, Harold Hamm Diabetes Center, University of Oklahoma Health Sciences Center, Oklahoma City, OK, United States

**Keywords:** endothelial function, adipose, liver, skeletal muscle, obesity, insulin resistance, diabetes, metabolism

## Abstract

Obesity is a worldwide epidemic that predisposes individuals to metabolic complications, such as type 2 diabetes mellitus and non-alcoholic fatty liver disease, all of which are related to an imbalance between food intake and energy expenditure. Identification of the pathogenic molecular mechanisms and effective therapeutic approaches are urgently needed. A well-accepted paradigm is that crosstalk between organs/tissues contributes to diseases. Endothelial dysfunction characterizes metabolic disorders and the related vascular complications. Over the past two decades, overwhelming studies have focused on mechanisms that lead to endothelial dysfunction. New investigations, however, have begun to appreciate the opposite direction of the crosstalk: endothelial regulation of metabolism, although the underlying mechanisms remain to be elucidated. This review summarizes the evidence that supports the concept of endothelial regulation of obesity and the associated insulin resistance in fat, liver, and skeletal muscles, the classic targets of insulin. Outstanding questions and future research directions are highlighted. Identification of the mechanisms of vascular endothelial regulation of metabolism may offer strategies for prevention and treatment of obesity and the related metabolic complications.

## Introduction

The triad of obesity, diabetes, and cardiovascular diseases (CVD) becomes a major public health threat because of the global shift toward an energy-rich and sedentary life style. The WHO predicts that overweight and obesity may soon become the most significant cause of poor health, in addition to malnutrition and infectious diseases (1). While half of the population in developed countries is overweight or obese, more than 15% of children in these countries are overweight or obese (1). Obesity and the associated metabolic complications, including metabolic syndrome and type 2 diabetes, contribute to cardiovascular morbidities and present a great threat to global human health (2). These complications account for more than 300,000 deaths per year in the U.S. (3, 4). Obesity results from an imbalance between energy intake and expenditure, which increases adipose tissue mass and ectopic fat accumulation. As a consequence, obesity leads to various defects, such as insulin resistance in fat, skeletal muscle, and liver, hepatosteatosis, pancreatic lipotoxicity, and eventually type 2 diabetes and other cardiovascular complications, including accelerated atherosclerosis (5). Identification of the pathogenic molecular mechanisms and effective therapeutic approaches are needed.

Despite decades of intense research, the major molecular pathogenesis behind complex metabolic diseases is unknown (6). A well-accepted paradigm is that cellular crosstalk in and between organs/tissues contributes to metabolic diseases (7–10). Endothelial dysfunction characterizes and contributes to the pathology of metabolic disorders and the related vascular complications (5, 11). Over the past two decades, overwhelming studies have focused on mechanisms that lead to endothelial dysfunction. The mechanisms include the reactive oxygen species-mediated eNOS uncoupling, loss of eNOS-derived NO bioavailability, hyperglycemia-promoted apoptosis of vascular endothelial cells (ECs) in diabetes, which ultimately leads to impaired endothelium-dependent vessel relaxation, a general biomarker of endothelial dysfunction (11–15). Emerging investigations, however, suggest that the opposite direction in the crosstalk, i.e., the endothelial regulation of metabolism, could be crucial, although the underlying mechanisms remain largely unknown. This review summarizes the emerging evidence that supports the concept of vascular endothelial regulation of obesity-associated insulin resistance in classic targets of insulin, including fat, liver, and skeletal muscles, and highlights the unanswered questions for future research directions. Advancement in understanding endothelial control of metabolism may provide novel approaches for intervention in obesity and obesity-related diseases.

### Endothelium and Endothelial Function

Endothelial cells form the lining of all blood and lymphatic vessels within the vascular network. The adult human body contains more than one trillion ECs, which weigh more than 100 g and cover a surface area of more than 1,000 square meters (16). As such, ECs can be regarded as an organ with endothelium forming a dynamic interface with all other organs in the body (17). Normal ECs line capillaries and establish specialized vascular niches or tissue-specific endothelium. On receiving cues from a wide variety of cells and tissues, this “organ” maintains vascular homeostasis by regulating each component of the endothelial function (18, 19). The most important endothelial function is to control vasomotor tone. The healthy ECs constitutively generate eNOS-derived NO to modulate vascular smooth muscle relaxation, which properly regulates both nutrient trafficking and blood fluidity. A well-controlled EC-derived NO is also essential to other aspects of the endothelial function, such as the capability of maintaining an anticoagulant, antiplatelet, and fibrinolytic phenotype of the endothelium (5). It has been well established that loss of endothelial function facilitates the development of atherosclerosis (20). Therefore, endothelium operates as a complex organ to maintain whole-body homeostasis by fulfilling proper endothelial function, which can be impaired under disease conditions, such as diabetes and obesity.

### Endothelial Dysfunction in Obesity and Insulin Resistance

Obesity contributes significantly to the development and progression of cardiovascular disease (21), which is believed to be mediated by oxidative stress, inflammation and endothelial dysfunction (22). Recent studies have highlighted the importance of metabolic pathways in ECs and suggested the therapeutic potential of targeting EC metabolism, since microcirculation is a key player in obesity-associated cardiovascular disease. Obesity elevates circulating free fatty acids and alters adipokines and inflammatory cytokines that are released from visceral and perivascular fat, which leads to structural and functional changes in microvasculature (23). Given the recognized differences in the metabolism of healthy ECs vs. diabetes-associated dysfunctional ECs, EC metabolism could be targeted for therapeutic benefit (24). For instance, by restoring diabetes-impaired glycolytic-influx and angiogenic response of the ECs, diabetic vasculopathies can be improved (24). Endothelial insulin resistance is associated with diabetic cardiovascular complications, including atherosclerosis (25). The role of endothelial insulin resistance in the development of obesity and systemic insulin resistance is not as straight-forward. Common treatments for obesity involving strategies to increase insulin sensitivity (26) have often been associated with improvement of endothelial function and/or endothelial insulin sensitivity (5). For instance, administration of metformin, which is known to enhance whole-body insulin sensitivity, improved endothelium-dependent vessel relaxation (27). Another excellent example is that physical activity has been found to modulate EC phenotype by overcoming obesity-induced endothelial dysfunction (28). However, emerging evidence support the concept of endothelial regulation of metabolism, e.g., through endothelial interactions with the insulin targets in obesity. Advancement in this research area may provide new strategies to prevent and treat obesity and obesity-related diseases.

## Endothelial Regulation of Obesity-Associated Insulin Resistance in Adipose Tissues

Obesity results from accumulation of white adipose tissue (29, 30). Adipose tissue is a central component for whole-body energy homeostasis. Insulin resistance in adipose tissue is presented as impaired insulin-stimulated glucose transport and blunted inhibition of lipolysis. However, adipocytes present a selective insulin resistance: while the insulin-promoted glucose transporter-4 trafficking is impaired, its impact on Forkhead box O-1 nuclear exclusion is preserved (31).

Responding to caloric intake, adipose tissue expands. People who are prone to deposit visceral but not subcutaneous adipose tissue have a higher risk of metabolic disease (32). Although normal subcutaneous adipose tissue expands with more capillary network than visceral tissue, this capability reduced with obesity and insulin resistance, suggesting that lack of angiogenesis in subcutaneous adipose tissue links to metabolic disease (33). The inability of peripheral adipose tissue to store excess energy has been reported in general population with insulin resistance (34, 35).

Linage tracing experiments demonstrated that the vascular endothelium of the adipose tissue gives rise to both white and brown fat cells (36), presumably through a Zfp423 (a multi zinc-finger transcription factor)-dependent pathway (37), suggesting a potential clinical use of EC-derived pre-adipocytes. In line with this, the capillary-derived beige adipocytes, when implanted in mice, improved metabolic homeostasis (38). Clearly, these data suggest that the plasticity and function of adipose tissue can both be regulated by proliferation/differentiation of stem cells and transformation of mature adipocytes by proper stimuli (39). It remains unknown, however, whether EC-derived factors are among these stimuli.

### Modulation *via* Angiogenesis

Adipose tissue angiogenesis and vascular functions have long been associated with obesity, adipose metabolism, and insulin sensitivity (40–42). Angiogenesis and adipogenesis are tightly coupled during development (43) and postnatal fat expansion (44). *In vitro* studies have shown that vascular-derived signals, e.g., endothelial-VEGF, directly affect the proliferation and differentiation of the surrounding pre-adipocytes, suggesting that angiogenesis can be a therapeutic target for obesity and metabolic diseases (45). Pharmacological inhibition of angiogenesis has been reported *in vivo* for the first time to reduce fat mass in distinct obesity models, likely through different vasculature-dependent mechanisms (46). However, the physiological consequences of modulating angiogenic activity seem to be context-dependent: gain of function studies show that overexpression of VEGF-A improves metabolic profiles, which maintains tissue function during the early phase of diet-induced obesity (44). In contrast, reducing vascularization promotes dysfunctional adipose cell death which may reverse obesity by improving metabolic outcomes in established obesity (47). Similar beneficial results were reported in nanoparticles-targeting adipose tissue transformation and angiogenesis, which prevented obesity in HFD-fed mice (48). It is unclear whether any EC-derived factors are involved in these studies.

### Modulation *via* Other EC-Derived Factors

Adipose stromal cells (ASCs) are therapeutically potent progenitor cells because they possess properties of pericytes. ASCs in combination with EC establish functional multilayer vessels. Transforming growth factor (TGF)-β is secreted by EC (49) and is able to induce αSMA expression in ASC, a marker of smooth muscle cell differentiation. A recent study found that ECs initiate this differentiation program in co-cultured ASC and propagate the program in distant ASC by induction of Activin A but not TGF-β (50, 51). Activin A is a secreted protein and a member of the TGF-β family that has pleiotropic effects on regulating apoptosis, proliferation, and cell differentiation (52). How ECs induce Activin A in ASC is yet to be determined (53).

Endocrine fibroblast growth factors (FGFs), such as FGF21, FGF15/19, and FGF23, are critical for maintaining whole-body homeostasis, because they operates in inter-organ endocrine signaling pathways that govern glucose, bile acid, and lipid metabolism (54, 55). FGF21 has gained much attention because of its favorable pharmacological properties in glucose and lipid metabolism (56). FGF21 is expressed in the liver, pancreas, adipose, and skeletal muscle, which can be altered by different stimuli, such as starvation or overfeeding (57). The role of endogenous FGF21 has just emerged (58). A recent study in HFD-fed mice (59) implicated that endothelial-derived FGF21 may contribute to improved metabolic profiles in mice lacking the autophagic protein LC3, although confirmative evidence is needed (e.g., demonstration of FGF21-dependency using FGF21-blocking antibodies (60) or endothelial FGF21 knockout mice). The role of autophagic proteins in the regulation of adipose tissue is not fully understood (61). If the role of LC3 is supported, it would be interesting and important to determine whether this feature also applies to other EC-derived FGFs.

### Modulation *via* MicroRNA (miRNA)

MicroRNAs recently link ECs to adipocyte function in diabetes and CVD (62, 63). One example is miR-181b, which was downregulated in adipose ECs from mice fed an HFD (64). Downregulation of miR-181b induced insulin resistance and low-grade inflammation in adipose tissue, which can be reversed by genetic delivery of miR-181b. Mechanistically, miR-181b targets the PH domain and leucine-rich repeat protein phosphatase-2 in ECs and, thus, improves eNOS-NO signaling. As such, adipose tissue ECs promote glucose uptake in adipocytes in a paracrine manner (64). miR-181b also decreased protein levels of vascular cell adhesion molecule and intercellular adhesion molecule, consistent with previous studies on systemic delivery of miR-181b (65, 66). Other miRNAs, such as miR-126, have been shown to preserve normal endothelial function, likely *via* blocking unwanted endothelial activation (67). These findings highlight the role of adipose ECs in the development of obesity-induced insulin resistance and the potential of using miRNAs as a tool to modulate EC function (68).

## Endothelial Regulation of Obesity-Associated Insulin Resistance in Skeletal Muscles

The skeletal muscle is one of the major target organs of insulin, essential for insulin-induced glucose uptake (69). Insulin resistance in skeletal muscle is manifested as a decrease in glucose transport and a decline in muscle glycogen synthesis in response to circulating insulin. Insulin sensitivity is decreased in myocytes obtained from obese individuals, or cultured myocytes in the presence of adipocyte-derived lipids (70), supporting the concept that accumulation of excess lipids or their metabolic derivatives causes decreased insulin signaling in skeletal muscle (71, 72). Insulin promotes the glucose uptake by increasing blood flow and recruiting perfused capillaries in skeletal muscle. Insulin signaling in vascular endothelium may control its own delivery to skeletal muscle and other tissues (73), although it remains to be determined whether this mechanism is central to systemic insulin sensitivity (74).

### Modulation *via* Vasodilation

Endothelial nitric oxide synthase (eNOS)-derived NO, the major endothelial-derived vasodilator, is central to endothelial regulation of insulin sensitivity in skeletal muscle, by stimulating blood vessel relaxation (in vascular smooth muscle cells) and perfusion (in skeletal muscles) (75). In addition, eNOS-derived NO regulates Akt signaling in skeletal muscle, through a cGMP-PI3K-dependent pathway (76). Interestingly, rapid formation of capillary ECs occurred in rat skeletal muscle after exposure to insulin (77), supporting the notion that insulin is a vascular hormone (78). However, the normal response of skeletal muscle capillary to NO is impaired in obesity-induced insulin resistance (79). Physical training appears to be able to improve it, likely because both endurance training and high intensity training with intervals increased eNOS protein contents specifically in the endothelium of capillaries and arterioles of skeletal muscle in previously sedentary lean and obese young men (80).

### Modulation *via* Barrier Function

There is evidence indicating that insulin delivery to skeletal muscle interstitium through the ECs is rate-limiting in insulin-stimulated glucose uptake in mice (74, 81) and humans (82). This process seems to be impaired by insulin resistance in type 2 diabetes and obesity (83). Mechanistically, damage to the endothelial glycocalyx barrier in skeletal muscle is believed to be an early event, as shown in mice fed an HFD (84). A recent study showed that endothelial insulin resistance also plays a pivotal role in the regulation of glucose uptake by the skeletal muscle (83). Mechanistically, HFD-impaired insulin signaling in EC downregulated insulin-induced eNOS phosphorylation and attenuated capillary recruitment and insulin delivery, which, in turn, reduced glucose uptake by the skeletal muscle (83). Conversely, restoration of the insulin-induced eNOS phosphorylation in ECs normalized capillary recruitment and insulin delivery, which restored glucose uptake by the skeletal muscle (81).

Fatty acids represent a key energy source that is used by a number of tissues, which must be tightly controlled to avoid lipotoxicity induced by an excess of unoxidized fatty acids (85). Obesity often causes over-accumulation of lipids in non-adipose tissues (e.g., skeletal muscle), which can trigger a toxic reaction and impair the function of the tissues, contributing to insulin resistance (86). Previous studies have identified endothelium as a key regulator of fatty acid transport, mediated by a complex signaling pathway including VEGF-B, PPARγ, and Apelin (87). In a recent study, endothelial Fcγ receptor IIB activation was shown to blunt insulin delivery to skeletal muscle, causing insulin resistance in mice (88). A study found that the capillary endothelial fatty acid binding proteins 4 and 5 (FABP4/5) are required for fatty acid uptake in heart and skeletal muscle, organs that possess muscle-type continuous capillary (89). In contrast, the function of an EC-derive mitogen activated protein kinase kinase kinase kinase 4 (MAP4K4) has opposing functions in blood and lymphatic ECs: while MAP4K4 is required for lymphatic vascular integrity, blood EC-MAP4K4 induces insulin resistance by impairing vascular function in obesity (90).

## Endothelial Regulation of Obesity-Associated Insulin Resistance in the Liver

The liver is responsible for maintaining fasting glucose levels. Excessive accumulation of lipids in the liver impairs hepatic responsiveness to insulin, leading to elevated levels of glucose and insulin in the circulation, eventually chronic hyperinsulinemia (91). Insulin resistance in the liver is selective in that insulin fails to suppress gluconeogenesis but continues to boost fatty acid synthesis (92). This uncoupling of insulin-mediated glucose and lipid metabolism will ultimately lead to hyperglycemia and hypertriglyceridemia, likely due to the unique activities of the mechanistic target of rapamycin complex (93) and the serine–threonine protein kinase (94) in hepatic lipogenesis. The role of endothelial regulation of insulin resistance in the liver has just begun to emerge.

### Liver Sinusoidal Endothelial Cells (LSECs) and the Pathology of Non-Alcoholic Fatty Liver Disease (NAFLD)

Obesity and insulin resistance can cause NAFLD, which is characterized by liver metabolic dysregulation, such as hyperlipidemia (95). Recent epidemiology of NAFLD and its connection with CVD suggest that blocking endothelial dysfunction would be beneficial (96), since endothelial function is impaired in patients with NAFLD (97).

Liver sinusoidal endothelial cells are highly specialized ECs, which form the wall of liver sinusoids and represent up to 20% of liver cells (98). LSECs are well known for their crucial roles in liver regeneration (99–101) and chronic liver diseases associated with obesity (e.g., fibrosis) (102). LSECs maintain hepatic stellate cells and hepatocytes in quiescence at basal condition but induce hepatocyte proliferation and angiogenesis during regeneration and undergo morphological and functional changes during fibrosis (103). It is believed that LSECs accomplish these by interacting with other hepatic cell types to maintain liver homeostasis. Since healthy LSECs facilitate the rapid transfer of fatty acids formed from cholesteryl esters to parenchymal cells, LSECs injury contributes to NAFLD (104). LSECs injury has been associated with NAFLD progression, where NAFLD may alter LSECs-mediated transport of nutrients, lipids, and lipoproteins. A recent study in choline-deficient, l-amino acid-defined, and high-fat diet models that mimic human NAFLD indicated that LSEC injury functions as “gatekeeper” in the progression from simple steatosis to the early non-alcoholic steatohepatitis stage, and LSEC injury may activate Kupffer cells and hepatic stellate cells, which in turn results in chronic liver injuries (105). In this regard, LSECs operate as a traffic-director at the crossroad of regeneration and fibrosis (106). It is yet to be determined whether LSEC-derived factors are required for this function.

### Intrahepatic Molecular Interactions

Iron overload often accompanies NAFLD. However, the key molecules involved in NAFLD-associated iron dysregulation have not been fully elucidated. A recent study found that LSEC secreted a bone morphogenetic protein-binding endothelial regulator that inhibited BMP-SMAD signaling in hepatocytes and reduced hepcidin protein expression (107). Fatty liver is the hepatic component of insulin resistance before developing NAFLD (108). In an attempt to identify physiological consequences of mice missing mucin-type *O*-glycans that are highly expressed in vascular ECs, a recent study found that the pups developed fatty liver disease, due to disconnected portal vein and intestinal lymphatic systems, leading to chylomicron deposition (109).

## Outstanding Questions and Future Directions

**Figure 1:**
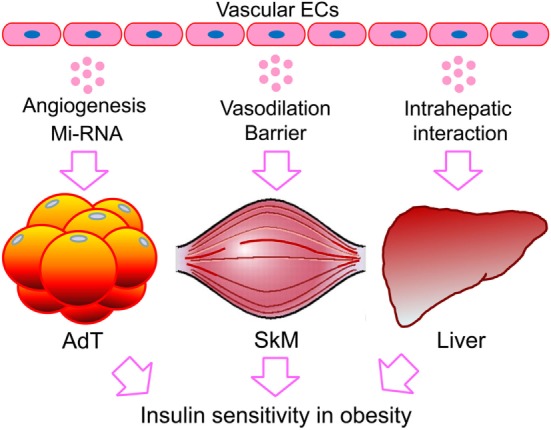
Endothelial regulation of obesity-associated insulin resistance in fat, skeletal muscle, and the liver. While direct interaction with ECs or EC-derived factors is expected to be shared by the classic targets of insulin, the other regulatory mechanisms could be tissue dependent. The preference in mode of action may reflect the distinct role of each organ/tissue in whole-body metabolism and the heterogeneity of the residing ECs. ECs, endothelial cells; miRNA, microRNA; AdTs, adipose tissues; SkM, skeletal muscle.

Our understanding of the endothelial layer has progressed significantly since its historical view as an inert layer of cells that serve as the inner lining of the circulatory system (110, 111). Now more than ever, the endothelium is demonstrated to regulate physiologic and pathologic processes by cross-talking with its residing organs. Over the past two decades, most research has focused on cellular and molecular mechanisms of endothelial dysfunction in disease conditions, such as diabetes (14, 112). Recent studies have looked into differences in the metabolism of healthy vs. diabetes in ECs, aiming at targeting endothelial metabolism for therapeutic benefit (24, 113). This review focuses on recent studies regarding the endothelial regulation of obesity-associated insulin resistance (Figure 1). The reviewed evidence supports an essential role for ECs in regulating the metabolic function of adipose tissues, skeletal muscle, and liver. However, except for the proposed regulation by EC-derived factors, other modes of regulation appear to be tissue-dependent. For instance, angiogenesis seems to be prominent in adipose tissue, consistent with its role in adipogenesis (21, 114). In contrast, blood vessel barrier function favors targeting the skeletal muscle, while LSEC intrahepatic interaction dominates in the liver (Figure 1). The difference may be ascribed to the distinct role of each organ/tissue in whole-body metabolism and the heterogeneity of the tissue-residing ECs. This concept may well apply to endothelial interaction with other type of cells not discussed here, such as inflammatory cells [e.g., macrophages (115)] and hypothalamic cells [e.g., hypothalamic-adipocyte axis (116) and brain (117)], which eventually affect insulin targets. The integrative conclusions of these studies present a picture of ECs interacting directly with cells of the classic insulin targets, through differential mechanisms, implicating organ- and pathway-specific imbalanced insulin actions (118). However, there are considerable unanswered questions that merit future investigations.

(1) While tissue- or organ-specific gene expression [e.g., tissue-specific promoters (119)] has been identified and widely used in pre-clinical studies, only global endothelial makers are currently available. To date, there are few reports on markers that may be unique to a tissue type [e.g., gene expression profile of microvascular ECs specific to the liver (120)] or to a vascular bed [e.g., a transcriptional factor restricted to arterial-ECs (121)]. Given the recognized phenotypic and functional heterogeneity in the endothelium, can we identify endothelial factors specific to their sites (e.g., tissues/organs or vascular beds) so that site-specific ECs could be manipulated for proof-of-concept studies and drug development?
(2) Phenotypic heterogeneity is a central feature of the endothelium, referring to differences in the location of the conduit vessels (e.g., arteries vs. veins) and their functions in serving specific organs (e.g., adipose tissue and skeletal muscle) (110, 111). In microvasculature, the diversity of the endothelium contributes to their specialized functions in regulating permeability, leukocyte trafficking, and hemostasis, the key features of endothelial function (122). How is this heterogeneity linked to the interactions between ECs and the classic targets of insulin discussed here?
(3) Endothelial cell-derived factors can be essential in maintaining organ homeostasis and regeneration. Could endothelium itself also operate as a rate-limiting apparatus [e.g., through its barrier function (123)] that regulates other hormones (e.g., IGFs and FGFs) in the same fashion as insulin for their delivery to targeted tissues or relevant locations?
(4) Severe impairment of endothelial function eventually promotes CVD. Non-invasive assessment of conduit vascular endothelial function is possible both in adults (124) and children (125) with metabolic disorders [e.g., using reactive hyperthermia and flow-mediated dilatation (126)]. In the same token, can endothelial regulation of metabolism be assessed *in vivo*? Or can specific parameters in this regard be identified by global assessment of gene expression profiles, epigenetic markers, and transcriptional factors?
(5) Over the past two decades, many experimental or therapeutic drugs that improve CVD risk factors also improved endothelial functions (127). Do these drugs modulate the crosstalk between ECs and the cells in their residing tissues? Or is this crosstalk an unrecognized mode of action, which should be considered in designing new and better drugs in the future?

In conclusion, the mechanisms underlying the endothelial-specific effects discussed here remain to be fully determined. Further translational studies to determine the clinical relevance of endothelial regulation of metabolism will provide greater insights that may ultimately lead to therapeutic advances against the increasing burden of the obesity pandemic and the associated metabolic disorders.

## Author Contributions

JX contributed to the conception. JX, ML, and MQ wrote the article.

## Conflict of Interest Statement

The authors declare that the research was conducted in the absence of any commercial or financial relationships that could be construed as a potential conflict of interest.
